# Review on generic methods for mechanical modeling, simulation and control of soft robots

**DOI:** 10.1371/journal.pone.0251059

**Published:** 2022-01-14

**Authors:** Pierre Schegg, Christian Duriez

**Affiliations:** 1 Robocath, Rouen, France; 2 Inria, CNRS, Centrale Lille, UMR 9189 CRIStAL, University of Lille, Lille, France; Scuola Superiore Sant’Anna, ITALY

## Abstract

In this review paper, we are interested in the models and algorithms that allow generic simulation and control of a soft robot. First, we start with a quick overview of modeling approaches for soft robots and available methods for calculating the mechanical compliance, and in particular numerical methods, like real-time Finite Element Method (FEM). We also show how these models can be updated based on sensor data. Then, we are interested in the problem of inverse kinematics, under constraints, with generic solutions without assumption on the robot shape, the type, the placement or the redundancy of the actuators, the material behavior… We are also interested by the use of these models and algorithms in case of contact with the environment. Moreover, we refer to dynamic control algorithms based on mechanical models, allowing for robust control of the positioning of the robot. For each of these aspects, this paper gives a quick overview of the existing methods and a focus on the use of FEM. Finally, we discuss the implementation and our contribution in the field for an open soft robotics research.

## 1 Introduction

Providing modeling and control methods for soft robots has been the topic of many recent works. This problem, which looked like a challenge a few years ago, now has technical solutions, even if they sometimes remain incomplete. The scope of this document is to provide a global view on model-based methods for soft robots. These robots being composed of deformable material, we are particularly interested in methods which account for a mechanical modeling of these deformations based on numerical schemes such as Finite Element Methods. We will recall that solutions exist to obtain real-time computation for these models. Then, we will examine how—once the bases of modeling posed—inverse modeling and control methods can be derived.

## 2 Soft robot modeling

Theoretically, soft robots have an infinite number of degrees of freedom. This makes them difficult to model analytically without strong assumptions. This section provides a quick review of modeling, from simplified analytical models to more generic, physics based, numerical models.

### 2.1 Analytical models

The main advantage of analytical models is that they are very fast to compute and can be derived easily for control law proofs. Their main limitation is their simplifying assumptions, which will be detailed in the following parts.

#### 2.1.1 Pseudo Rigid Body Model

Rod-like robots can be modeled using the Pseudo-Rigid Body Model. Using this method, continuous elongated robots, such as steerable catheters used in surgical robotics, are approximated by a n-link rigid manipulator with a torsional spring in each link. This model assumes there is only bending and no torsion in the robot [1, 2].

#### 2.1.2 Constant Curvature Model

The Constant Curvature Model is a simplifying approach to allow closed form kinematics and enable computing the Jacobian of a soft continuum robot. The main assumptions are that there is zero torsion—meaning that the deflection is always planar—and that the bending section always takes the shape of a circular arc—constant curvature [3].

The main advantage of this type of modeling is that it can be derived into Denavit-Hartenberg parameters, Frenet-Serret frames, an integral representation or exponential coordinates. This allows to use control laws adapted from rigid robotics.

Piecewise constant curvature models are used for robots shaped by continuously bending actuators or tendons, concentric tube robots, and steerable needles. Initially, they were the most used in the Soft Robotics literature [4]. Examples of applications include a cable-driven octopus arm [5] and a pneumatic soft gripper [6]. Though this model has been extended to handle dynamics [7] it still has major shortcomings. Especially this model is not valid if the robot is subject to external forces which are out of its plane, such as gravity [8].

### 2.2 Numerical models

Numerical models discretize the geometry into a mesh and use a numerical solver, which always makes approximations. Numerical models are, in theory, less precise than analytical models but in the case of continuum mechanics for deformable solids, it’s often the opposite. As there is no analytical solution for most of the cases, analytical models are only used in continuum mechanics for very simplified shapes, constitutive laws and boundary conditions, far from the deformations obtained on soft robots.

#### 2.2.1 Cosserat rod theory

To address some of the shortcomings of the models presented before, physics based models of deformation were proposed. Cosserat geometrically exact models for instance, based on Cosserat rod theory, were developed to model these same elongated robots with a much improved accuracy [9] and in a mathematical framework close to rigid robot models (local coordinates and integration of motions using the Lie group).

Using Cosserat models, deformable objects are represented as a space curve and a coordinate frame of director vectors is associated to each point. These models can handle kinematics and dynamics of objects undergoing large deformations such as threads in surgical simulations [10] and of various soft robots, ranging from an octopus arm [11] to underwater bio-inspired robots [12, 13]. The model is based on the deformation of a center line and sections perpendicular to this line are considered rigid. Therefore, one limitation is that not all deformations are taken into account. In addition, while the model simplifies the expression of strain forces, complex numerical approaches are needed to integrate the dynamics. But this approach makes possible to physically model the deformations, in particular by taking into account the constitutive law of the materials.

#### 2.2.2 Finite element modeling

As illustrated in previous parts, it is difficult to obtain an analytical mechanical model for soft robots and some models that keep an analytical description of the geometry, like Cosserat rods, are limited to specific shapes of robots. Moreover, most of these models, do not yet model contacts precisely.

The Finite Element Method (FEM) is a numerical method for solving Partial Differential Equations, which can be used to model soft robots [14]. This method can handle generic shapes and constitutive materials. It can also model contact interactions between the soft robot and other objects in the environment, soft or rigid [15]. This method is very well known and widely used in industry, in particular for simulating multiphysics. It is very appealing in the design process of complex systems (such as soft robots), with a easy loop between CAD and numerical tests. The Finite Element Method was shown to be the most realistic in simulating soft robots mechanics as it describes non-linearities better and can model interactions between different materials within the same robot [16, 17].

The main drawback of using this method is its computation cost. Various methods have been developed to model and simulate soft deformable objects in real time, for instance using GPU computation in the context of haptic rendering [18, 19]. Fast implementation of the Finite Element Method to model soft and deformable objects are abundant in the Computer Graphics community [20, 21] and in the Surgical Simulation community [18, 22, 23]. Examples of applying this method to soft robots include [14, 24–31], and to haptic devices [19, 32]. FEM can also be leveraged to optimize the shape and mechanical properties of a soft robot or its actuation [33–35].

Another promising method for speeding up the simulation is Model Order Reduction [36, 37]. Using Model Order Reduction, Thieffry has shown the simulation is fast enough to use it for dynamic control [30, 38, 39] (see also section 3.4). Finally, recent works in surgical simulation explore using machine learning to learn the deformations of soft organs and tissues [40, 41].

### 2.3 Online update of the models

Whatever the model used for the robot, an objective in robotics is to be able to update it according to the information retrieved from sensors placed on the robot. In rigid robotics, for example, a sensor is placed on the joints to measure their position and update the kinematic model. In soft robotics, the difficulty is that the system theoretically has an infinite number of degrees of freedom and it is therefore theoretically impossible to measure the complete state of the system.

However, in practice, analytical models described above and Cosserat-type models have a relatively small state, which for instance allows using visual servoing to update the model [42, 43] or more localized sensing like multi-magnet tracking [44]. Resistive, capacitive, inductive or optical sensors have been also developed more widely in soft robotics [45] and are usually coupled with analytical models to couple the physical measure to the deformation, like in [46] for capacitive sensing. However, it is often difficult to combine information from various types of sensors with these models.

FEM models allow multi-physics coupling and such a model can be used to integrate or merge multi-sensor information [47]. However, with these numerical models, the state of the robot is usually much larger and can not be fully sensed. In [26], a condensation of the FEM model is used to write a kinematic model of the soft-robot in a reduced space. The full model is then updated through visual servoing on a reduced number of feature points of the robot, allowing closed loop control (see also section 3.2.2). This approach is extended in [48] to capture deformations and deduce external loads applied on a soft structure. An other possible strategy for FEM is to use model-order reduction to obtain a good dynamic model with a reduced size. Then, it is shown in [39] that the information obtained from a magnetic tracking system can update a state-observer in a context of dynamic control (see also section 3.4).

Despite this work, which shows significant progress, the updating of soft robots models based on a sensor system remains a very open problem.

## 3 Soft robot control

### 3.1 Control methods using Forward Kinematics

#### 3.1.1 Direct control

In the case of direct control, forward kinematic models can be used to validate a robot’s design, as a proof of concept, to study the robot’s controllability, and to optimize the placement of sensors. Examples of direct control of soft robots include a pneumatic actuated snake-like robot [49] and a caterpillar inspired robot actuated by shape memory alloy actuators [50]. Both use periodic electrical wave signals inputted to the actuators, generating different gaits.

#### 3.1.2 Learning based control

As soft robots can be tricky to model, a tempting solution is to adopt a “model-free” approach and learn the control policy directly [51]. Several Reinforcement Learning algorithms have been used for continuum robot control (such as catheter navigation in medical robotics [52]) and soft robotics control. Applications include manipulation and navigation tasks. A recent survey of these applications to soft robotics can be found in [53]. A subset of these applications can be found in the following table.

**Table:** 

	Application	Algorithm used
Navigation of an autonomous robot	Reach destination based on first person view and target image [54]	A3C
	Navigating on a path while avoiding pedestrians [55]	PPO
	Simultaneous Localisation and Mapping [56]	Neural SLAM
	Cognitive Mapping and Planning [57]	DAGGer
Soft robotics manipulation	Door opening [58]	Modified NAF
	Screwing a bottle, inserting a block in a hole [59]	Extended GPS
	Predicting optimal control [60, 61]	MPC + GPS

### 3.2 Control methods using Inverse Kinematics and Inverse Dynamics

#### 3.2.1 Analytical methods

Analytical solutions require a low computational cost and often reliably offer a global solution. In the case of rigid robotics, robots are often designed to guarantee there exists a solution to the inverse kinematics problem. For soft robots, obtaining such guarantees on an analytical inverse kinematics model requires strong assumptions on the robot’s geometry. As an example, for rod-like robots, the Constant Curvature Model or the Piecewise Constant Curvature Model provide analytical Inverse Kinematics Solutions [4]. Application examples include steerable concentric tubes [62] and a multi section continuum trunk [63].

#### 3.2.2 Jacobian based

In the context of rigid robotics, the Jacobian matrix is defined as the differential relationship between actuator variables and end-effector position. One popular way to solve the inverse kinematics problem is to invert this matrix. With some specific assumptions, this Jacobian matrix can be extended to soft robotics.

In the example of a conical manipulator driven by cables [43], the Jacobian matrix can be defined as the relationship between the 2D position of the robot’s tip and the cable tensions. Authors then propose a control method based on the Jacobian inverse to solve the Inverse Kinematics problem.

Assuming that the robot’s workspace has no singular configuration, that the actuators are not constrained and that they can build a FEM-based simulation which is an observer of the robot, Zhang and coauthors define a FEM-based Jacobian matrix and design a closed-loop controller for a parallel soft robot using Jacobian pseudo-inverse [26, 64].

To control a deformable needle inside soft tissues, the Finite Element method is also used to compute a Jacobian matrix of the coupled system and the Jacobian pseudo-inverse [65, 66].

#### 3.2.3 Optimization based methods

*3.2.3.1 Problem formulation*. The Inverse Kinematics problem can be formulated as a constrained optimization problem. This formulation can handle a large number of degrees of freedom, and singularities can be handled by adding constraints. This framework also handles actuator constraints and many design variables can be included. This formulation is widely used in rigid robotics and computer graphics [67]. Numerous ways of solving such constrained optimization problems have been developed.

*3.2.3.2 Non linear programming*. A constrained optimization problem is non linear if either the objective function or at least one constraint is a non linear function. In the computer graphics community, constrained optimization has been used to compute muscle actuation to animate a face [68], or optimize actuator placement and the material configuration of deformable objects [20] and plush toys [21], as well as the actuation to obtain given deformations.

*3.2.3.3 Quadratic programming*. If the objective function has a quadratic form and the constraints are linear, then the constrained optimization problem is a Quadratic Program.

In the Computer Graphics community, such a formulation has been used for instance to model and animate tendons and muscles in human hands. Given the motion of the bones, they use Quadratic Programming to compute the corresponding muscle activation and the force transmission via the tendons [69].

In the context of surgical simulation, a Quadratic Program is used to register the movements and deformations of deformable glands due to weight loss during radiotherapy [23].

Finally, Quadratic Programming is applied to the control of soft robots using cable and pneumatic actuation in [70] and the results are validated using simulated and real robots. The same control algorithms have also been used to create haptic interfaces [32] and have been extended to use hydraulic actuators [71]. Finally, using two simulations running in parallel, a closed loop controller can be designed. The first simulation is computing the Inverse Kinematics and is used to calculate the actuation. The second simulation, also based on a quadratic program, uses the information of sensors, computes the Forward Kinematics and is used as a state estimator [27].

#### 3.2.4 Learning based methods

Recently, Machine Learning techniques have shown great success in regression problems, essentially learning a mapping between input features and output features. It is possible to create a dataset of robot configurations and postures. If one has access to accurate simulations, this process can be sped up by running several simulations in parallel to generate the data. A Machine Learning algorithm is then trained to learn the Inverse Kinematics of the robot, meaning the mapping between the actuator variables and the robot pose. The main advantage of these methods is that they are very fast to provide a solution to the IK problem, and thus enable real time control. This method has been extensively studied for computer graphics [67] and rigid robotics [72].

*3.2.4.1 Feed forward neural network*. Several works have used feed-forward neural networks to learn the mapping between the end-effector pose and the actuator variables. This method has been applied to controlling a soft robot based on Inverse Dynamics [73]. It has also been applied to the control of a soft arm [74, 75] and soft manipulators [76, 77].

*3.2.4.2 Gaussian Process Regression*. Another way to learn the IK or ID mapping is to rely on statistical methods such as Gaussian Process Regression. The computer graphics community have used these methods to generate natural looking poses of humanoid figures [78, 79]. Gaussian Process Regression has also been applied to rigid robotics [72] and most recently to soft robots to learn the modeling error [80] or the Inverse Kinematics used for control [81].

### 3.3 Inverse Kinematics with contacts handling

One of the main advantages of soft robots over rigid ones is their intrinsic safety when interacting with the environment. Thus most use cases of deformable robots involve contacts between the robot and its surroundings. Control strategies should therefore account for contacts.

#### 3.3.1 Jacobian based

A model-less approach can be used for controlling a continuum manipulator’s end effector position [82]. The robot’s Jacobian is first estimated offline by moving each actuator in increments and observing the resulting end effector displacement. The Jacobian is then updated online using measurements of actuators and end effector positions and solving a constrained optimization problem. This control algorithm can handle environments with unknown obstacles.This work was extended to also handle force control [83].

#### 3.3.2 Optimization based

Optimizing trajectories of rigid systems with a large number of links and actuators can be done using Quadratic Programming. One of the key aspects is including the contacts in a way that doesn’t render the search space discontinuous or too local minima prone. [84] proposes to include the contact forces as decision variables in the optimization and use a complementarity formulation. [85] models contacts as continuous variables. Using these strategies they are able to generate complex motions of several simulated rigid link mechanisms.

In Computer Graphics, a common problem is that of moving a deformable object in a realistic manner. Automating part of this animation process requires modeling the physics of the objects and the contacts with the environment. Several works use penalty-based contact models and solve a Quadratic Program to optimize the shape of the deformable characters and generate plausible motions [86, 87].

In the case of soft robots, external contacts will create deformations that will potentially modify the robot kinematic relationship between inputs and outputs. There is therefore a total coupling of the contacts with the inverse model of the robot. In this case, Signorini’s law can be used to model contacts and a Quadratic Program with Complementarity Constraints can be built. This method has been demonstrated on several robots, both simulated and real, for navigation, locomotion and manipulation tasks. This framework also computes the IK at interactive rates [24, 88].

### 3.4 Dynamic control

Kinematic controllers are sometimes not sufficient to perform high speed tasks, or to compensate for vibrations for instance. To tackle these issues dynamic controllers have been designed.

#### 3.4.1 Existing approaches and state of the art

To manage the dynamics, Quadratic Programming can still be used by using a dynamic model of the soft robot. This makes it possible, offline, to plan its trajectory. An open-loop approach is presented in [29]. In [89], a Sequential Quadratic Program is used to solve the inverse dynamics of a fluid powered soft manipulator and iterative learning control is leveraged to perform trajectory optimization.

In order to perform closed loop control, real time performance has to be obtained. Several closed loop controllers based on the constant curvature or piecewise constant curvature models extended to dynamics have been proposed. Authors of [90] use the PCC hypothesis to describe their soft robot with Denavit-Hartenberg parameters and then use a dynamic controller designed for rigid robotics to control their system. This work has been continued using synergistic control and an extended controller from rigid robotics [91].

In [92], authors use the Koopman operator to construct a linear model of a soft arm actuated by pressure. Using this linear model they can perform Model Predictive Control by iteratively solving a quadratic program.

Based on a Cosserat model for statics and a Lagrangian model for dynamics [93], use Ritz and Ritz-Galerkin approaches to design a dynamic controller for a continuum manipulator and compares the results with several other models.

As stated before these models make the hypothesis of no deformation of the robot section, only the centerline of the robot deforms. This makes it possible to have models that are very quick to calculate.

In a more general design case, the use in real time of a FEM type numerical model, even if calculated in real time, is not fast enough for a real time control of the dynamics (the calculation must be faster than the eigenfrequencies of the robot). However, by applying Model Order Reduction techniques on FEM models, classical tools of control theory can be used to control the dynamical behavior of soft robots in simulation [30], and on real robots actuated by cables [39, 94] or pneumatics [95]. Stability can also be guaranteed using Lyapunov theory [38] with a proof of robustness to modeling errors generated by the reduction. This work was extended to use a reduced non linear model of the robot by leveraging a Linear Parameter Varying model [31].

#### 3.4.2 Current limitations and challenges

Dynamic models can be used offline for trajectory calculation. By using dynamic models with few state variables (whether simplified or reduced), it is possible to build low-level controllers for robots with guaranteed robustness. Recent progresses in this challenge of dynamic control of soft robots are really impressive.

However, for performance reasons, these approaches do not yet integrate a correct management of contacts and interactions with the environment, which is essential for robot dynamics.

Moreover, the challenge ahead is undoubtedly to increase the performances of soft robots that are still poor. It can be done by combining mechanical design and control strategies. These two being generally separated.

## 4 Implementation

This paper presents several existing methods to model and control soft robots but emphasizes the use of real-time FEM. This section focus on existing implementation of this approach.

### 4.1 SOFA

To model and simulate robots using the Finite Element Method, we use the free and open source software SOFA [96]. SOFA uses the theory of continuum mechanics for the material modeling, constraints are solved using Lagrange multipliers and contact interactions are handled using Signorini’s law [24]. Internal forces are computed based on a user chosen deformation law. Several deformation laws are available: linear options such as Hook’s law, as well as non linear strain-stress relationships or plastic deformations for instance. Several plugins are also available to extend SOFA’s functionalities for multiple applications, ranging from medical simulation to haptics. Some of these plugins are useful to soft robotics and will be described further.

### 4.2 SoftRobots and SoftRobots.Inverse plugins

SOFA was first intended for medical simulation. The SoftRobots plugin was introduced in 2017 to model and simulate deformable robots and their environment in SOFA [25]. The plugin takes advantage of the model of continuum mechanics and the Finite Element Method implemented in SOFA to apply them to simulating deformable robots. It also combines previous works on inverse FEM simulation [22, 32], soft robot control [14, 97] and real time simulation [18]. The SoftRobots.Inverse plugin allows for optimization-based inverse model control of soft robots based on Quadratic Programming, and can handle contacts [24].

### 4.3 Model Order Reduction

As expressed before, one of the main issues with the Finite Element Method is the computation cost. When using this method, the user must compromise some accuracy to be able to achieve real time simulation. This can take the form of using a coarser mesh, or a larger simulation timestep.

To address this issue, several approaches have been proposed to reduce the model. The objective here is to find a model with fewer variables which faithfully describes the behavior of the full order model according to some measure. Note that different measures may lead to different reduced models.

A plugin for Model Order Reduction is available in SOFA [36]. Using this plugin, the simulation can be made fast enough for dynamic control [30, 31, 38, 39, 94]

### 4.4 Hardware platforms

In order to test the algorithms, several hardware platforms have been designed. These platforms aim to test a broad variety of actuators and to represent a diversity of use cases. Most of these robots are presented in [70] and [24].

**Fig 1:**
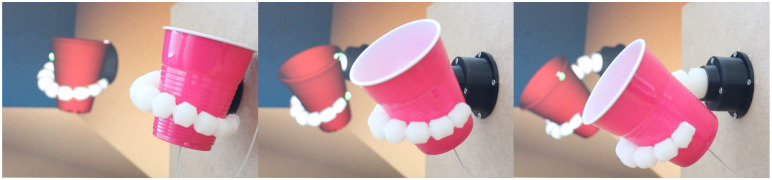
Soft gripper based on an elephant trunk holding a deformable cup.

The most recent results include holding and manipulating a deformable cup with a silicone elephant trunk using Quadratic Programming [88] and a dynamic closed loop controller based on a reduced model of this same robot was proposed in [39, 94] to cancel the vibrations. Authors also compare open loop and closed loop control. A view of this robot holding a cup can be found in Fig 1.

**Fig 2:**
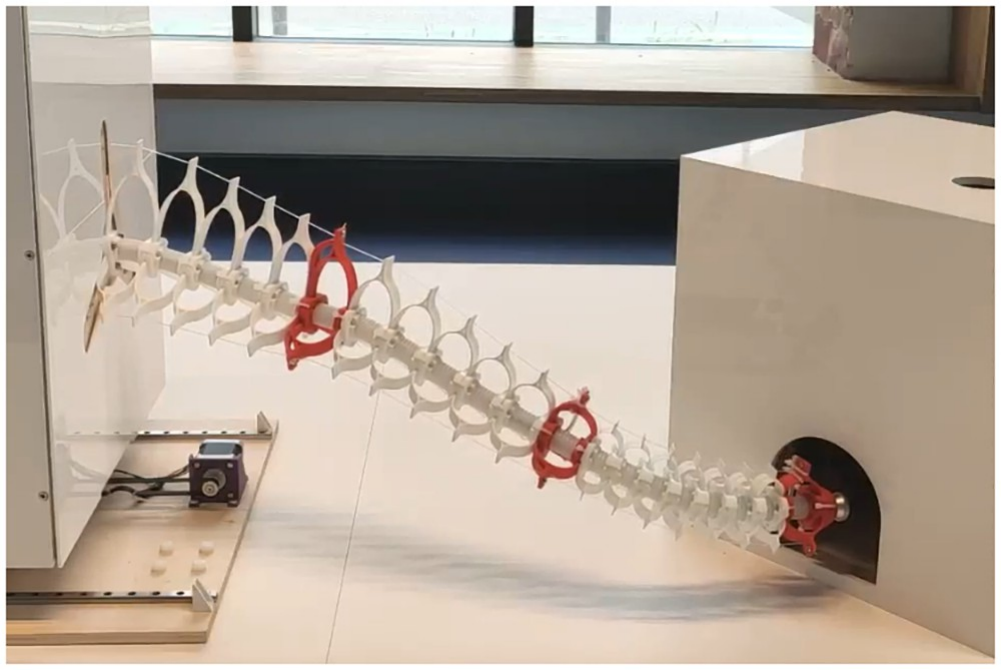
The Echelon 3 soft manipulator.

Authors of [28] show the full pipeline starting from the design of a soft manipulator called Echelon 3, then modeling and simulating it using FEM. This allows to derive forward and inverse models, used to design open loop and closed loop controllers. These controllers are then experimentally validated and compared on the physical prototype and a haptic feedback loop is created. A picture of the physical prototype can be found in Fig 2.

## 5 Conclusion

The last decade has seen huge progress on low level control of soft robots, to the point that it is now no longer a question of whether we can control a specific soft robot, but to what degree of precision.

Performance of soft robots are not yet to the level of rigid robots though, and some work still remains to be done on design optimization, design of controllers especially dynamic ones and sensors.

Thanks to the use of models of soft robots for low level control, the higher level control and decision making policies are very close to those already implemented for rigid robotics or other fields such as autonomous driving. Indeed we can now use trajectory optimization or Model Predictive Control on soft robots.

In this context, the usefulness of FEM and more generally of physics-based numerical models computed in real time, has been demonstrated: it unifies the modelling from design to control and allows interactions with the environment to be taken into account. In the future, these methods will probably be coupled with learning approaches to correct modeling errors, to complete the control with higher-level decisions and to obtain more automatically the numerical model of the robot in its environment.
